# Improving the Catalytic Property of the Glycoside Hydrolase LXYL-P1–2 by Directed Evolution

**DOI:** 10.3390/molecules22122133

**Published:** 2017-12-04

**Authors:** Jing-Jing Chen, Xiao Liang, Hui-Xian Li, Tian-Jiao Chen, Ping Zhu

**Affiliations:** State Key Laboratory of Bioactive Substance and Function of Natural Medicines; Key Laboratory of Biosynthesis of Natural Products of National Health and Family Planning Commission, Institute of Materia Medica, Peking Union Medical College & Chinese Academy of Medical Sciences, 1 Xian Nong Tan Street, Beijing 100050, China; chenjingjing@imm.ac.cn (J.-J.C.); liangxiao@imm.ac.cn (X.L.); lihuixian@imm.ac.cn (H.-X.L.); chentianjiao@imm.ac.cn (T.-J.C.)

**Keywords:** glycoside hydrolase, directed evolution, error-prone PCR, catalytic efficiency, 7-β-Xylosyltaxanes

## Abstract

The glycoside hydrolase LXYL-P1–2 from *Lentinula edodes* can specifically hydrolyze 7-β-xylosyltaxanes to form 7-β-hydroxyltaxanes for the semi-synthesis of paclitaxel. In order to improve the catalytic properties of the enzyme, we performed error-prone PCR to construct the random mutant library of LXYL-P1–2 and used the methanol-induced plate method to screen the mutants with improved catalytic properties. Two variants, LXYL-P1–2-EP1 (EP1, S91D mutation) and LXYL-P1–2-EP2 (EP2, T368E mutation), were obtained from the library and exhibited 17% and 47% increases in their catalytic efficiencies on 7-β-xylosyl-10-deacetyltaxol. Meanwhile, compared with LXYL-P1–2, EP1 and EP2 showed elevated stabilities in the range of pH ≥ 6 conditions. After treatment at pH 12 for 48 h, EP1 and EP2 retained 77% and 63% activities, respectively, while the wild-type only retained 33% activity under the same condition. Molecular docking results revealed that the S91D mutation led to a shorter distance between the R-chain and the substrate, while the T368E mutation increased negative charge at the surface of the enzyme, and may introduce alterations of the loop near the active pocket, both of which may result in improved stabilities and catalytic activities of enzymes. This study provides a practical directed evolution method for exploring catalytically improved glycoside hydrolase.

## 1. Introduction 

Paclitaxel (Taxol^®^) refers to a diterpenoid compound with definite anti-cancer effect. Due to its clinical application, paclitaxel has become one of the most important anti-tumor drugs [1,2,3]. With the development of its new indications and formulations, its clinical application has also expanded. Both the domestic and overseas market demand for paclitaxel is still increasing, although the natural concentrations in yew plants is extremely low [4,5]. 7-β-Xylosyltaxanes, such as 7-β-xylosyl-10-deacetyltaxol (XDT), are the analogues of paclitaxel produced by yew plants (*Taxus* species), which are generally discarded during paclitaxel extraction, leading to both resource waste and potential environmental pollution [4,6]. In previous studies, our group cloned and characterized a new β-xylosidase (designated as LXYL-P1–2) from *Lentinula edodes*, which can specifically hydrolyze XDT to form 10-deacetyltaxol (DT) [7]. DT can be used for the semi-synthesis of paclitaxel. Moreover, the β-xylosidase is heterologously expressed successfully in *Pichia pastoris* [7]. In addition, we also have conducted scaling-up of high-cell-density fermentation of the recombinant yeast (up to 1000-L fermenter), which can be used to convert XDT into DT [8,9,10]. In view of the application prospect of this glycoside hydrolase in the pharmaceutical industry, it is promising to explore variants with higher catalytic activity, thus providing the important significance for industrial applications. 

Directed evolution is a method used in protein engineering that allows natural evolutionary processes to be mimicked in the laboratory. It is considered as a useful tool for studying the relationship between the sequence and function of an enzyme, which is also an efficient method for optimizing biocatalysts for practical applications [11,12,13,14,15]. The key step in performing directed evolution is the generation of a library of variant genes, which lays the foundation for subsequent selection. In most cases, directed evolution requires a large number of mutants, such as a reservoir capacity of 10^3^ to 10^6^ mutants [14]. The construction process of the mutant library usually involves the following steps: at first, random point mutations in a population of DNA products are introduced through using an approach, such as the error-prone PCR [16]. Controlling the concentrations of Mg^2+^, Mn^2+^ and dNTP in the PCR mixture can influence the mutation frequency [17,18]. Secondly, the mutated genes are ligated with the expression vector by applying efficient cloning technology such as In-Fusion cloning method [19]. Thirdly, the recombinant expression plasmids are transformed into the suitable host, such as *E. coil* and *P. pastoris*, generating the mutant library. The majority of mutations generated in this way are deleterious, so libraries of mutants tend to mostly have variants with reduced activity [12]. Therefore, a reliable and effective screening system is needed to find the rare variants with desired properties [20,21]. To date, there have been reported successes in improving the activity, specificity, or tolerance to extremes of pH or temperature of enzymes by directed evolution [22,23,24,25,26,27,28,29]. For example, endo-β-1, 4-xylanase mutants with improved catalytic efficiencies were obtained through using error-prone PCR and a high-throughput screening system. Compared with the wild-type enzyme, the catalytic efficiencies of two variants were increased by 25% and 89%, respectively [25]. Moreover, the thermostability of the endo-β-1, 4-xylanase from *Thermomyces lanuginosus* was improved by directed evolution using error-prone PCR [28]. Hence, directed evolution is particularly suited for improving enzymes by generating promising variants in a rapid and effective way. 

The glycoside hydrolase LXYL-P1–2 belongs to the GH3 family of glycoside hydrolases. Glycoside specificity analysis suggests that LXYL-P1–2 exhibits both β-d-xylosidase and β-d-glucosidase activities [7]. In the present study, we employed a directed evolution approach with the aim to improve catalytic effectiveness of the glycoside hydrolase. By using error-prone PCR coupled with a 96-well plate screening system, two variants with improved catalytic properties were obtained from the random mutant library. The β-xylosidase activities of the recombinant yeasts and purified enzymes were significantly increased compared with those of the wild-type. The present study offers a practical directed evolution method to generate catalytically improved glycoside hydrolase. 

## 2. Results

### 2.1. Establishment of the Methanol-Induced Plate Screening Approach for Mutants with Improved Enzyme Activities

**Figure 1:**
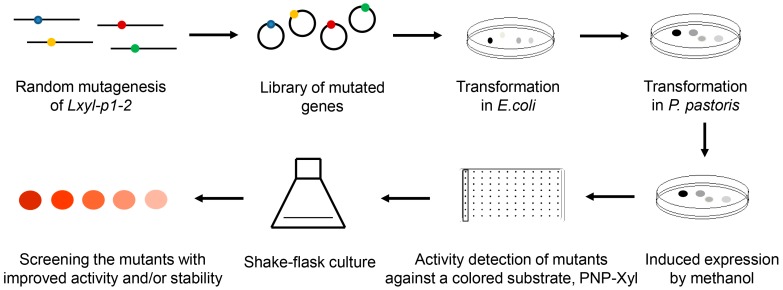
Flow chart of the screening process.

As outlined in Figure 1, the random point mutations were introduced into the *Lxyl-p1–2* gene by error-prone PCR, and the PCR fragments were ligated with expression vectors based on In-Fusion cloning method. Then the recombinant plasmid libraries were transformed into *P. pastoris*, and the LXYL-P1–2 mutant library was successfully generated. In order to ensure the induction expression of the glycoside hydrolase, each single colony was cultured on the plate with 1% methanol, and 200 μL of methanol was further added to the plate cover every day. As the glycoside hydrolase was expressed intracellularly in the recombinant yeasts, the change in enzymatic activity was detected by monitoring the β-xylosidase activity of cells in vivo. Based on these, a reliable 96-well plate screening system was established to obtain the mutant enzymes with improved activity. Approximately 1200 colonies were primarily screened, most of which were with reduced activities, accounting for 85.6% of the total screening clones. A portion of them were with unchanged activities, occupying 14.2% of the total screening clones. Only two variants exhibiting higher catalytic efficiencies than the wild type strain GS115-3.5K-P1–2 (GS115-P1–2), were referred to as GS115-3.5K-P1–2-EP1 (GS115-EP1) and GS115-3.5K-P1–2-EP2 (GS115-EP2). 

### 2.2. β-Xylosidase Activities of the Recombinant Yeast GS115-EP1 and GS115-EP2 against PNP-Xyl 

**Figure 2:**
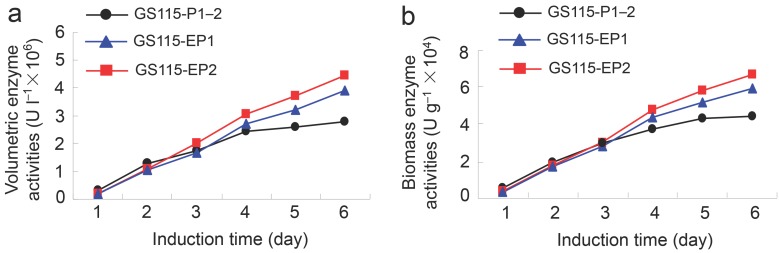
β-Xylosidase activities of the recombinant yeasts GS115-EP1 and GS115-EP2 against PNP-Xyl. (**a**) Volumetric enzyme activities; (**b**) biomass enzyme activities.

The two recombinant yeast strains GS115-EP1 and GS115-EP2 were cultured in a shake flask and the protein expressions were induced by methanol for five days. The volumetric enzyme activities and the biomass enzyme activities of the strains were determined as described previously [10]. As shown in Figure 2, with the increase of induction time, the volumetric activities and biomass activities of wild-type strain and mutant strains were increased. After induction by methanol for three days, the enzyme activities of GS115-EP1 and GS115-EP2 exceeded that of the wild type GS115-P1–2, of which the activity of GS115-EP2 increased more effectively. The volumetric activities of GS115-EP1 and GS115-EP2 were achieved 3.93 × 10^6^ U L^−1^ and 4.45 × 10^6^ U L^−1^, respectively, at the induction time of five days, which are increased by 40% and 59% compared with that of GS115-P1–2 (control) (2.8 × 10^6^ U L^−1^). Similarly, the biomass activities of GS115-EP1 and GS115-EP2 reached 5.96 × 10^4^ U g^−1^ and 6.71 × 10^4^ U g^−1^, respectively, which are increased by 34% and 51% in comparison with that of the control (4.45 × 10^4^ U g^−1^).

### 2.3. Conversion Rates of 7-β-Xylosyltaxanes by the Recombinant Yeasts GS115-EP1 and GS115-EP2 

After five days of cultivation, the methanol induced recombinant cells were harvested and used as a biocatalyst to convert 7-β-xylosyltaxanes mainly containing XDT (62.12%), 7-β-xylosyl-10-deacetylcephalomannine (XDC, 12.75%) and 7-β-xylosyl-10-deacetyltaxol C (XDTC, 17.04%). After reaction for 24 h at 45 °C, most of the substrates were converted to corresponding products. As shown in Table 1, the conversion rates of XDC, XDT, and XDTC by the recombinant yeast GS115-EP1 were 1.15, 1.09, and 1.11 times as high as that of the wild-type, respectively. Similarly, the conversion rates of XDC, XDT, and XDTC by the recombinant yeast GS115-EP2 were 1.16, 1.09, and 1.13 times as high as that of the wild-type, respectively. The results suggest that both the variants could more efficiently convert 7-β-xylosyltaxanes compared with the wild-type.

### 2.4. Sequencing Analysis of EP1 and EP2

The genomic DNA of GS115-EP1 and GS115-EP2 was extracted and the *Lxyl-p1–2* variants were amplified. DNA sequencing results indicated that *Lxyl-p1–2-EP1* (*EP1*) had three nucleotide substitutions: T^271^→G^271^, C^272^→A^272^, and A^273^→T^273^, resulting in the amino acid S91D mutation. *Lxyl-p1*–*2-EP1* (*EP2*) had three nucleotide substitutions: A^1102^→G^1102^, C^1103^→A^1103^, and T^1104^→A^1104^, which led to T368E mutation. 

### 2.5. Characterization of Recombinant Proteins EP1 and EP2

**Figure 3:**
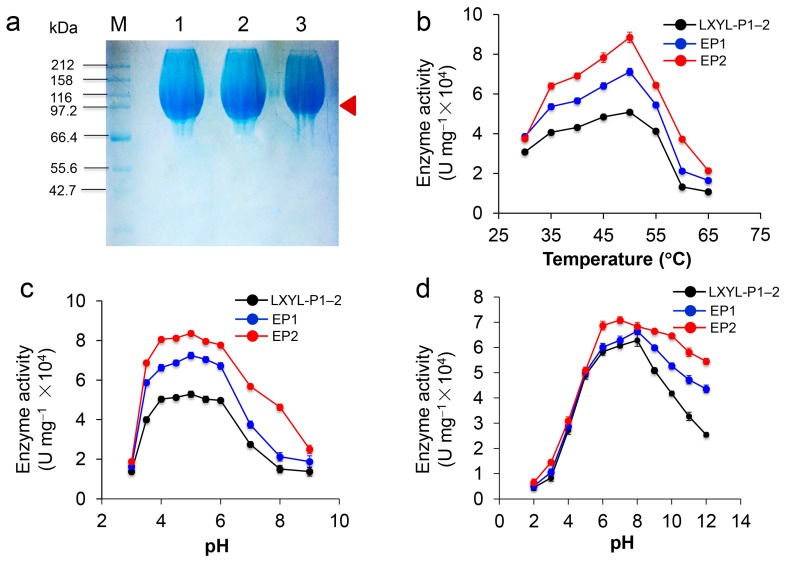
Characterization of the recombinant EP1 and EP2 against PNP-Xyl. (**a**) SDS-PAGE analysis. The arrow indicates the band of the recombinant enzyme. M, the protein molecular marker; 1, 2, 3 indicate the purified recombinant LXYL-P1–2, EP1, and EP2 (glycosylated proteins) prepared by gel column chromatography; (**b**) Effect of temperature on β-xylosidase activity against PNP-Xyl; (**c**) Effect of pH on β-xylosidase activity against PNP-Xyl; (**d**) Effect of pH on the stability of LXYL-P1–2, EP1 and EP2 (50 mM acetate, PBS, or Tris-HCl buffers, 4 °C).

The recombinant proteins were purified by Ni-NTA affinity chromatography and gel column chromatography. SDS-PAGE analysis demonstrates that the purified recombinant LXYL-P1–2, LXYL-P1–2-EP1 (EP1), and LXYL-P1–2-EP1 (EP2) are highly glycosylated with molecular weights of 100–115 kDa (theoretical molecular weight is about 82 kDa), and each of them has a dispersion phenomenon in the electrophoresis band (Figure 3a). To explore the enzymatic activity of EP1 and EP2, the enzyme-catalyzed reactions were conducted at different temperatures and pHs. As shown in Figure 3b,c, similar to LXYL-P1–2, the optimum temperature of EP1 and EP2 against PNP-Xyl was 50 °C. The suitable reactive pH of the two enzymes ranged from 4.0 to 6.0, with an optimum pH of 5.0. Besides, the pH stability of EP1 and EP2 was further examined. After the enzyme was incubated in different buffers with pH ranging from 2.0 to 12.0 for 48 h, the catalytic reactions against PNP-Xyl were conducted at 50 °C, pH 5.0 for 20 min. The results showed that the enzyme EP1 was most stable at pH 8.0, which is similar to LXYL-P1–2, and the enzyme EP2 was most stable at pH 7.0. Moreover, both EP1 and EP2 were able to tolerate harsh alkaline conditions, retaining 77% and 63% of their highest enzyme activity after exposure to pH 12.0 for 48 h. In contrast, the wild type only retained 36% of its maximal activity under the same condition. The results showed that the tolerance of the two enzymes at pH 6.0~12.0 were higher than that of the control, of which EP2 was much more stable under the alkaline conditions (Figure 3d). 

### 2.6. Characterization and Kinetics of the Recombinant Proteins EP1 and EP2

The specific activities of the two variants against PNP-Xyl and PNP-Glc were tested. The results showed that the β-d-xylosidase and β-d-glucosidase activities of EP1 and EP2 were both higher than that of LXYL-P1–2. Compared with LXYL-P1–2, the β-d-xylosidase and β-d-glucosidase activities of EP1 were increased by 40.35% and 39.5%, respectively, while the β-d-xylosidase and β-d-glucosidase activities of EP2 were increased by 61.44% and 31.29%, respectively.

The kinetic parameters of EP1 and EP2 against XDT were also measured, and the results can be found in Table 2 and Table 3. The *K*_m_ of LXYL-P1–2 against XDT was approximately three-fold higher than those of EP1 and EP2, indicating that the affinities of EP1 and EP2 to the substrate XDT were significantly increased (** *p <* 0.01). However, the turnover numbers (*k*_cat_) of EP1 and EP2 were lower than that of LXYL-P1–2. Altogether, compared with that of LXYL-P1–2, the catalytic efficiencies (*k*_cat_*/K*_m_) against XDT of EP1 and EP2 were increased by 17.2% and 46.6%, respectively (** *p <* 0.01). The results suggest that the single point mutation at the position 91 or 368 of the glycosidase could lead to the increased affinity of the two mutant proteins to the substrate, resulting in a higher catalytic efficiency and conversion rate of XDT. In addition, the above results also conform to the results at the cell level, as shown in Table 1. 

### 2.7. Three-Dimensional Structure Prediction of Enzyme

**Figure 4:**
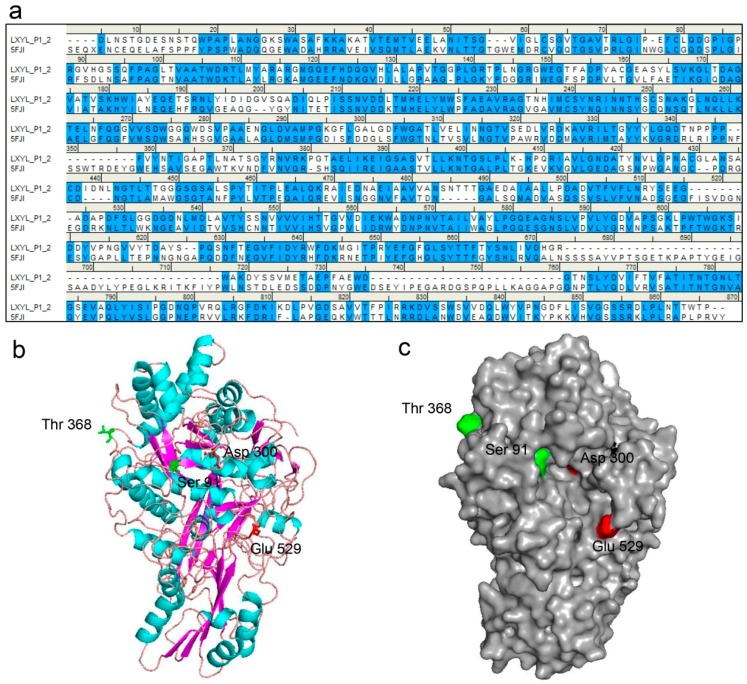
The LXYL-P1–2 structure predicted by homology modeling. (**a**) Sequence alignment between LXYL-P1–2 and the template GH3 β-glucosidase from *A. fumigatus* (PDB 5fji). Identical and similar residues are highlighted in blue; (**b**) Three-dimensional structure view. The β-strands are represented as magenta, α-helices as cyan, and loops are colored in salmon; (**c**) Surface view. The catalytic sites (Asp^300^, Glu^529^) are indicated in red, the substitutions occurred in the mutants EP1 and EP2 are indicated in green. The homology model of LXYL-P1–2 was constructed based on the template GH3 β-glucosidase from *A. fumigatus* (PDB 5fji) using SWISS-MODEL.

The molecular structure of LXYL-P1–2 was predicted by molecular modelling (http://swissmodel.Expasy.org/). By a protein–protein BLAST, the GH3 β-glucosidase from *Aspergillus fumigatus* (Protein Data Bank code 5fji) is the most similar enzyme among the GH family, which exhibited 44% sequence identity and 59% sequence similarity to the glycoside hydrolase LXYL-P1–2. Therefore, it was selected as the template for LXYL-P1–2 homology modelling. The sequence alignment between LXYL-P1–2 and the template is shown in Figure 4a. Moreover, as shown in Figure 4b, the predicted structure of the wild type enzyme had the canonical three-domain structure feature belonging to the GH3 family. The N-terminal (α/β)_8_-barrel domain houses the nucleophile (Asp^300^) whilst the α/β-sandwich domain houses the catalytic acid (Glu^529^), which was supported by previous prediction and point mutation validation [7]. In addition, the single mutated amino acid in position 91 of the mutant EP1 is situated within the N-terminal domain, and closed to the nucleophile Asp^300^. In contrast, the mutation in position 368 of EP2 is located on the loop and at the surface of the predicted protein (Figure 4b,c). The T368E substitution increases the negative charge of the mutant protein, which may lead to the improved stability at a higher pH value.

### 2.8. Substrate Docking Analysis of Mutants EP1 and EP2

**Figure 5:**
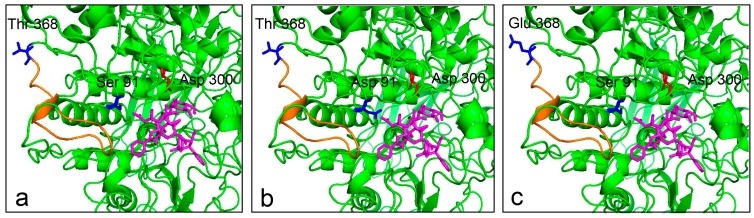
Molecular docking of wild type and mutants with XDT. XDT is shown in magenta. The nucleophile Asp^300^ is colored in red. The side chains of Ser^91^/Asp^91^ and Thr^368^/Glu^368^ are shown in blue. (**a**) Side view of LXYL-P1–2 with XDT, showing Ser^91^ and Thr^368^; (**b**) Side view of EP1 with XDT, in which Ser^91^ is replaced by Asp^91^; (**c**) Side view of EP2 with XDT, in which Thr^368^ is replaced by Glu^368^. The alteration of the loop near the active pocket is indicated in orange.

To identify the possible molecular basis for the improvement of catalytic property, a docking model of the enzyme-XDT complex was constructed based on the homology model (Figure 5). The docking results indicated that the residue Ser^91^ in the β-sheet is located in the near extension path of the XDT binding region, while the side chain of the substituted Asp^91^ extends further into the active pocket, making the stronger interaction between the mutant protein and XDT possible. The mutation of T368E is in the loop of the enzyme near the active pocket, potentially leading to the increased affinity to the substrate.

## 3. Discussion

The disadvantages of poor activity and stability limited the applications of some enzymes, directed evolution technique can be used to improve enzyme specific properties, and it has acquired great success in changing enzyme characteristics [12,24]. The glycoside hydrolase LXYL-P1–2 from *L. edodes* has a great potential in bioconverting 7-β-xylosyltaxanes from agriculture wastes into valuable products in pharmaceutical industries [7]. In the present study, we employed a directed evolution technique to enhance the catalytic efficiency of LXYL-P1–2.

The directed evolution strategy of LXYL-P1–2 involves the random mutagenesis of *Lxyl-p1*–*2* gene, followed by a screening or selection progress to screen or enrich for the variants with improved enzyme properties. In our previous study, we optimized the error-prone PCR conditions including the concentrations of Mg^2+^ and Mn^2+^ to control the mutation frequency, and also optimized the ligation efficiency of In-Fusion cloning method to obtain the recombinant plasmids efficiently [30]. The highest ligation rate was around 90% when the homologous length reached 100 bp, which was two to three times higher than those of the homologous length of 15 bp (recommended by the manufacturer’s instructions) and the conventional restriction and ligation method. Subsequently, the recombinant LXYL-P1–2 mutant library was successfully generated. PNP-Xyl can be hydrolyzed into xylose and *p*-nitrophenol, and the content of *p*-nitrophenol can be measured by the absorbance at 405 nm. Therefore, the activity of mutant enzymes of LXYL-P1–2 can be rapidly measured by using the chromogenic substrate PNP-Xyl. The mutated enzymes were expressed intracellularly in the recombinant yeasts [6,7,9], which allowed us to readily distinguish the change in enzymatic activity by monitoring the β-xylosidase activity of recombinant cells in vivo. Finally, based on the error-prone PCR coupled with a methanol-induced plate screening approach, two positive mutants—GS115-EP1 and GS115-EP2—with improved properties were selected (Figure 1).

Our results showed that, after five-day induction, the recombinant yeasts GS115-EP1 and GS115-EP2 apparently surpassed the wild type control both on the volumetric and biomass activities (Figure 2). Consequently, the conversion rates of 7-β-xylosyltaxanes by the recombinant yeast GS115-EP1 and GS115-EP2 were also increased compared with that of the control (Table 1). Furthermore, we purified the mutant proteins EP1 (S91D) and EP2 (T368E). Although optimum reaction pH and temperature of the two enzymes were the same as those of LXYL-P1–2, the stabilities at alkaline conditions of the two mutants, especially EP2, were higher than that of the control. The increased tolerance on the wide ranges of pH may facilitate the manipulation of the reactions with combined enzymes [31]. Substitution of the amino acids on the surface of the protein has effects on its stability depending on the environment of the mutation sites [25]. Compared with the wild type, EP2 had a glutamic acid residue instead of threonine residue at the position of 368. Since threonine is a polar uncharged amino acid while glutamic acid is an acidic amino acid, the substitution of glutamic acid for threonine may protect the protein core from the OH^−^ attack, and maintain its enzymatic activity at a higher alkaline pH value. 

In addition, we also measured the specific activity of the two variants against PNP-Xyl and PNP-Glc as well as the enzyme kinetics against XDT. The results showed that the specific activity, the affinity and catalytic efficiency (*k*_cat_/*K*_m_) against XDT of the two mutant enzymes were significantly higher than that of LXYL-P1–2. Substitutions of S91D and T368E resulted in 67% decrease in *K*_m_ value on XDT and 17–47% increases in their catalytic efficiencies (*k*_cat_*/K*_m_) (Table 2 and Table 3). Regarding the *K*_m_ apparent values, the two mutants displayed improved apparent affinity for XDT. Homology modeling and molecular docking can be used to elucidate how the mutations affect catalysis and enzyme stability [32]. The models derived from GH3 β-glucosidase from *A. fumigatus* enabled us to propose a possible mechanism that led to the significant increase in the substrate affinity of the mutant. Molecular docking results showed that Ser^91^ was located in the near extension path of the XDT binding pocket and closed to the nucleophile Asp^300^ in the catalytic center of the enzyme (Figure 4 and Figure 5). When the Ser^91^ was substituted with Asp^91^ in EP1, the distance between the R-chain of the aspartic acid and the substrate was closer, which may further facilitate the interaction between the enzyme and the substrate (Figure 5). The amino acid alteration in EP2 was found at the surface region of the protein far away from the catalytic region but located in the loop present in the enzyme, part of which extended near the outside of the active pocket. The single mutation T368E does not seem to change the overall architecture of the enzyme but may introduce alteration of the loop that indirectly promotes interaction between the enzyme and the substrate. These results indicate that the 368 site plays an important role in the enzyme property although it is far away from the active pocket, as has been reported elsewhere [33]. 

In summary, we have established a methanol-induced plate screening approach which can be employed to obtain glycoside hydrolase LXYL-P1–2 mutants with improved enzyme properties. Engineered yeast with LXYL-P1–2 variants could convert 7-β-xylosyltaxanes into 7-β-hydroxyltaxanes with higher yields. Moreover, the two mutants were determined to be S91D and T368E mutations, and both mutations apparently increased the enzyme affinity to XDT. The results show that the mutations of S91D and T368E improve enzyme properties including the β-xylosidase activity and the alkaline stability. These findings are beneficial for the bioconversion of 7-β-xylosyltaxanes for the semi-synthesis of paclitaxel, and have practical significance for the application of glycoside hydrolase in the pharmaceutical industry. 

## 4. Materials and Methods 

### 4.1. Plasmids and Strains

*Lxyl-p1*–*2* from *L. edodes* M95.33 was cloned into pPIC3.5K and the resulting expression vector was designated pPIC3.5K-LXYL-P1–2 used as the PCR template. *Pichia pastoris* GS115-3.5K-P1–2 was constructed by transforming the host strain *P. pastoris* GS115 (Mut^+^) with the plasmid pPIC3.5K-LXYL-P1–2, and was then preserved at −80 °C prior to use [7].

### 4.2. Error-Prone PCR and Construction of the Library of Variant Lxyl-p1–2 Genes

To construct a library of *Lxyl-p1*–*2* variants, mutations were randomly introduced using error-prone PCR. The vector pPIC3.5K-LXYL-P1–2 containing *Lxyl-p1–2* gene was used as the DNA template. The sense primer *Lxyl-p1–2*-100F (5′-TTTCATAATTGCGACTGGTTCCAATTG-3′) and antisense primer *Lxyl-p1–2*-100R (5′-CTGACATCCTCTTGATTAGAATCT-3′) were used for the gene fragment amplification, resulting in the amplified fragment containing the 100 bp extensions at each 5′ and 3′ end that were homologous to the ends of the linearized vector pPIC3.5K. The PCR mixture (50 μL) was composed of 10 × *Taq* buffer, 10 μM primers (*Lxyl-p1–2*-100F and *Lxyl-p1–2*-100R), 10 ng template plasmid pPIC3.5K-LXYL-P1–2, 2.5 units of Taq DNA polymerase, 1 mM or 0.2 mM dATP and dCTP, 1 mM or 0.2 mM dTTP and dGTP. In addition, 0.5 mM MnCl_2_ and 7 mM MgCl_2_ were also added into the PCR mixture to increase the mutation frequency. The PCR reaction was carried out in an thermocycler (Eppendorf, Hamburg, Germany) under the conditions as follows, 94 °C for 5 min, 30 cycles of 94 °C for 30 s, 55 °C for 30 s, 72 °C for 2 min, and 72 °C for 10 min. The PCR products were isolated from a 1.0% agarose gel and purified using a Qiaquick Gel Extract kit (Transgen, Beijing, China). The DNA fragments were treated by *Dpn* I to remove the template, and the expression vector pPIC3.5K was digested by *Bam* HI and *Not* I (NEB, Ipswitch, MA, USA). Then the PCR segments were subsequently ligated into the linearized vector at the molar ratio of 5:1 using eFusion cloning kit (Biophay Biotechnology Co. Ltd., Beijing, China). Finally, the ligation products were transformed into *E. coli* TG1 and placed onto LB-ampicillin plates for screening of mutants. The recombinant plasmids of all the transformants were extracted using the Prep Mini plasmid kit (Transgen, Beijing, China), and further transformed into the *P. pastoris* GS115 via electroporation transformation according to the manufacturer’s instructions (Invitrogen, Carlsbad, CA, USA), resulting in recombinant yeast cells that carried the various *Lxyl-p1–2 mutated* genes. 

### 4.3. Screening of Mutants with Higher β-Xylosidase Activities

The recombinant yeasts harboring the *Lxyl-p1–2* variants were cultivated on the BMMY plate (contents per liter: 10 g yeast extract, 20 g peptone, 100 mM potassium phosphate buffer, pH 6.0, 10 mL methanol, and 15 g agar) at 30 °C for 2–3 days, and 200 μL methanol was added to the plate cover every day for the induction of the gene expression. Then each single colony was picked into a 96-well plate containing 100 μL of 5 mM *p*-nitrophenyl-β-d-xylopyranoside (PNP-Xyl, Sigma, St. Louis, MO, USA) as chromogenic substrate. After 20 min of incubation at 50 °C, the reaction was stopped by adding 500 μL of stop solution of saturated sodium tetraborate (Na_2_B_4_O_7_). The reaction mixtures were centrifuged at 3000 rpm for 10 min, and 200 μL of the supernatants were transferred to another 96-well plate. Subsequently, the absorbance at 405 nm was measured using spectrophotometry (NanoPhotometer^®^ P300, IMPLEN, Munich, Germany) for the β-*xylosidase* activity analysis. The yeast GS115-3.5K-P1–2 was used as the control. As the increased absorbance indicates the increased activity, the mutants with increased absorbance (compared to the control) were screened for further enzyme activity analysis.

### 4.4. Measurement of β-Xylosidase Activity of Mutant Strains

The screened variants were further inoculated into 500 mL shaking flasks, each containing 100 mL of the BMGY medium (similar to BMMY but contained 10 mL L^−1^ glycerol instead of 10 mL L^−1^ methanol). The flasks were incubated at 30 °C and 220 rpm for 24 h, and methanol was added every day to maintain 1% (*v*/*v*) for the induction of the gene expression. Meanwhile, the β-xylosidase activity was analyzed on the basis of periodic sampling. The samples were washed twice with dH_2_O via centrifugation, and the cell pellet was resuspended with dH_2_O in the same volume of the culture broth. 10 μL of the cell suspension was added to 100 μL of 5 mM PNP-Xyl, and incubated for 20 min at 50 °C for the catalytic activity analysis. The β-xylosidase activity was then evaluated by calculating both U L^−1^ (volumetric enzyme activity) and U g^−1^ (biomass enzyme activity) as described previously [10]. Furthermore, the bioconversion of 7-β-xylosyltaxanes by the recombinant yeast mutant was also measured. The yeast cells induced by methanol for five days were harvested. The wet cells (65 mg mL^−1^) were used to convert 10 mg mL^−1^ of crude XDT extract (XDT, 62.12%; 7-β-xylosyl-10-deacetylcephalomannine, 12.75%; 7-β-xylosyl-10-deacetyltaxol C, 17.04%) (provided by Fujian South Biotechnology Co., Mingxi, China). The reaction was performed in 50 mM sodium acetate buffer at 45 °C for 24 h. The products were extracted with ethyl acetate and the yields were assayed via HPLC as described previously [7]. The conversion rate was calculated as product peak area/(substrate peak area + product peak area) × 100%.

### 4.5. Sequencing Analysis of Lxyl-p1–2 Variants 

The genomic DNA of mutants with higher β-xylosidase avtivities was extracted via TIANamp Yeast DNA Kit (TIANGEN, Beijing, China) and used as the PCR template. The standard primer 5′AOX1 and 3′AOX1 were used to amplify the *Lxyl-p1–2 variants*. Then the amplified fragment was cloned into the pEASY-T1 vector (Transgen, Beijing, China). It was then sequenced using a DNA sequencer (ABI PRISM^®^ 3100 Genetic Analyzer, Applied biosystems, Foster City, CA, USA). The DNA sequences were analyzed compared with the wild type parent using the DNAMAN software, (Version 6.0, Lynnon Corporation, San Ramon, CA, USA). 

### 4.6. Enzyme Purification and SDS-PAGE Analysis

The enzymes of the mutants were isolated and purified based on our previous report [7]. Briefly, 5 g freeze-dried yeast cells were suspended in 50 mL Tris-HCl buffer (20 mM, pH 8.0) and subjected to 10 cycles of high-pressure cell disruption (APV-2000, SPX Corporation, Charlotte, NC, USA) at 1200 bar, 4 °C. The supernatant was obtained by centrifugation at 16,000 *g* and 4 °C for 30 min. After gradient elution of different concentration of imidazole (buffer A: 20 mM, pH 8.0; buffer B: 60 mM, pH 8.5; buffer C: 200 mM, pH 8.0) by nickel bonded affinity chromatography, samples eluted from buffer B were collected and concentrated (UFC903096 30KD, Millipore, Billeri, MA, USA). 10 μL of the concentrated samples were mixed with 2.5 μL of sample buffer (5× sample buffer, Invitrogen, Carlsbad, CA, USA) and incubated for 5 min at 100 °C. Then, SDS-PAGE was performed using a 5% stacking gel and a 12% separating gel on a vertical mini gel apparatus (Bio-Radmini-2D, Bio-Rad, Hercules, CA, USA), as described by Laemmli46. Protein molecular weight marker was purchased from NEB (P7702, Ipswitch, MA, USA). The purified recombinant LXYL-P1–2 was prepared previously by our lab and was used as the control. Gels were stained with Coomassie Brilliant Blue R-250 (Sigma Chemical, St. Louis, MO, USA).

### 4.7. Characterization and Kinetics of the Recombinant LXYL-P1–2 Mutants

Determination of the optimum reaction temperature of the mutants on PNP-Xyl: A total reaction mixture of 125 μL contained 5 mM PNP-Xyl and 25 μL of 0.10 mg mL^−1^ purified LXYL-P1–2 mutant enzymes in 50 mM sodium acetate buffer with pH 5.0. The reaction was performed under the following temperatures: 30, 35, 40, 45, 50, 55, 60, and 65 °C for 20 min.

Determination of the optimum reaction pH of the mutants on PNP-Xyl: a total reaction mixture of 125 μL contained 5 mM PNP-Xyl and 25 μL of 0.10 mg mL^−^^1^ purified LXYL-P1–2 mutant enzymes in 50 mM sodium acetate buffer with pH 3.0, 3.5, 4.0, 4.5, 5.0, and 5.5; or in 50 mM potassium phosphate buffer with pH 6.0 and 7.0; or in 50 mM Tris-HCl buffer with pH 8.0 and 9.0. The reaction was performed under 50 °C for 20 min. 

Determination of pH stability of the mutants: the enzyme was properly dissolved in the various 50 mM buffer solutions including sodium acetate buffer (pH 2.0–5.0), potassium phosphate (pH 6.0–7.0), and Tris-HCl (pH 8.0–12.0) at the concentration of 0.1 mg mL^−1^, and the solutions were incubated at 4 °C for 48 h. Then the enzyme solutions were mixed with 5 mM PNP-Xyl, and the reaction was performed under 50 °C for 20 min. 

Enzymatic activity analysis: the β-d-xylosidase and β-d-glucosidase activities were measured by detecting the amount of *p*-nitrophenol released from the substrate PNP-Xyl or PNP-Glu under the optimum reaction conditions. 60 μL reaction volume contained 50 μL of 5 mM PNP-Xyl/PNP-Glc and 10 μL of 0.1 mg mL^−1^ enzyme in 50 mM sodium acetate buffer with pH 5.0. The reaction was performed under 50 °C for 20 min. Reactions were terminated by adding 2 mL saturated Na_2_B_4_O_7_. The enzymatic activity was assayed using spectrophotometry based on the absorbance at 405 nm. One unit of activity was defined as the amount of enzyme that catalyzed the formation of 1 nM *p*-nitrophenol per minute. The kinetic parameters of the recombinant mutant enzymes against XDT were determined in the XDT concentration range of 0.039–5.0 mM as described previously [7]. DT formation was analyzed via HPLC. The kinetic data on XDT were processed via a proportional weighted fit using a nonlinear regression analysis program based on Michaelis–Menten enzyme kinetics. All data are presented as means ± SD of three independent repeats.

### 4.8. Homology Modeling and Molecular Docking 

The three-dimensional model of glycoside hydrolase LXYL-P1–2 was built by SWISS-MODEL Server (http://swissmodel.expasy.org/) [34] using the available structure of the *Aspergillus fumigatus* (Protein Data Bank code 5fji), which has 44% sequence identity or 59% sequence similarity with LXYL-P1–2, as template. Predicted structure and the substrate XDT were selected for the docking experiments with AutoDockTools.

## Figures and Tables

**Table 1:** Conversion rates of 7-β-xylosyltaxanes and the yields by the recombinant yeast strains.

Name	Conversion Rates (%)			Yields (mg mL^−1^)			Total Yields
	XDC	XDT	XDTC	DC	DT	DTC	(mg mL^−1^)
GS115-P1–2	75.78	76.77	76.97	0.61	3.02	0.82	4.45
GS115-EP1	87.41	83.95	86.01	0.67	3.27	0.89	4.83
GS115-EP2	87.68	83.74	86.87	0.68	3.33	0.91	4.92

Notes: XDT, 7-β-xylosyl-10-deacetyltaxol; DT, 10-deacetyltaxol; XDC, 7-β-xylosyl-10-deacetylcephalomannine; DC, 10-deacetylcephalomannine; XDTC, 7-β-xylosyl-10-deacetyltaxol C; DTC, 10-deacetyltaxol C.

**Table 2:** Specific activities of EP1 and EP2 against PNP-Xyl and PNP-Glc.

	PNP-Xyl		PNP-Glc	
	U mg^−1^ (×10^4^)	Relative Activity (%)	U mg^−1^ (×10^4^)	Relative Activity (%)
LXYL-P1–2	5.03 (±0.14)	100.00	12.05 (±0.49)	100.00
EP1	7.06 (±0.56) **	140.35	16.81 (±0.53) **	139.50
EP2	8.12 (±0.63) **	161.44	15.82 (±0.85) **	131.29

Note: *n* = 3, ** *p <* 0.01 vs. control (LXYL-P1–2).

**Table 3:** Kinetic parameters for the hydrolysis of XDT by EP1 and EP2.

	*V*_max_ (μmol L^−^^1^ min^−1^)	*K*_m_ (mmol L^−^^1^)	*k*_cat_ (s^−^^1^)	*k*_cat_/*K*_m_ (mmol L^−^^1^ s^−^^1^)
LXYL-P1–2	8.16 (±0.15)	0.51 (±0.01)	4.89 (±0.10)	9.67 (±0.10)
EP1	3.15 (±0.06)	0.17 (±0.01) **	1.89 (±0.04)	11.33 (±0.39) **
EP2	3.84 (±0.05)	0.16 (±0.01) **	2.30 (±0.03)	14.17 (±0.81) **

Note: *n* = 3, ** *p <* 0.01 vs. control (LXYL-P1–2).
